# Prostate Cancer Peripheral Blood NK Cells Show Enhanced CD9, CD49a, CXCR4, CXCL8, MMP-9 Production and Secrete Monocyte-Recruiting and Polarizing Factors

**DOI:** 10.3389/fimmu.2020.586126

**Published:** 2021-01-25

**Authors:** Matteo Gallazzi, Denisa Baci, Lorenzo Mortara, Annalisa Bosi, Giuseppe Buono, Angelo Naselli, Andrea Guarneri, Federico Dehò, Paolo Capogrosso, Adriana Albini, Douglas M. Noonan, Antonino Bruno

**Affiliations:** ^1^Laboratory of Immunology and General Pathology, Department of Biotechnology and Life Sciences, University of Insubria, Varese, Italy; ^2^Laboratory of Pharmacology, Department of Medicine and Surgery, University of Insubria, Varese, Italy; ^3^Unit of Immunology, IRCCS MultiMedica, Milan, Italy; ^4^Unit of Urology, San Giuseppe Hospital, IRCCS MultiMedica, Milan, Italy; ^5^S.C. of Urology, ASST Settelaghi, Ospedale di Circolo e Fondazione Macchi, Varese, Italy; ^6^Laboratory of Vascular Biology and Angiogenesis, IRCCS MultiMedica, Milano, Italy

**Keywords:** natural killer cell, myeloid cells, monocytes, macrophages, immune cell polarization, inflammation, angiogenesis, prostate cancer

## Abstract

Natural killer (NK) cells, effector lymphocytes of the innate immunity, have been shown to be altered in several cancers, both at tissue and peripheral levels. We have shown that in Non-Small Cell Lung Cancer (NSCLC) and colon cancer, tumour associated circulating NK (TA-NK) and tumour infiltrating NK (TI-NK) exhibit pro-angiogenic phenotype/functions. However, there is still a lack of knowledge concerning the phenotype of peripheral blood (PB) NK (pNK) cells in prostate cancer (PCa). Here, we phenotypically and functionally characterized pNK from PCa patients (PCa TA-NKs) and investigated their interactions with endothelial cells and monocytes/macrophages. NK cell subset distribution in PB of PCa patients was investigated, by multicolor flow cytometry, for surface antigens expression. Protein arrays were performed to characterize the secretome on FACS-sorted pNK cells. Conditioned media (CM) from FACS-sorted PCa pTA-NKs were used to determine their ability to induce pro-inflammatory/pro-angiogenic phenotype/functions in endothelial cells, monocytes, and macrophages. CM from three different PCa (PC-3, DU-145, LNCaP) cell lines, were used to assess their effects on human NK cell polarization *in vitro*, by multicolor flow cytometry. We found that PCa pTA-NKs acquire the CD56^bright^CD9^+^CD49a^+^CXCR4^+^ phenotype, increased the expression of markers of exhaustion (PD-1, TIM-3) and are impaired in their degranulation capabilities. Similar effects were observed on healthy donor-derived pNK cells, exposed to conditioned media of three different PCa cell lines, together with increased production of pro-inflammatory chemokines/chemokine receptors CXCR4, CXCL8, CXCL12, reduced production of TNFα, IFNγ and Granzyme-B. PCa TA-NKs released factors able to support inflammatory angiogenesis in an *in vitro* model and increased the expression of CXCL8, ICAM-1, and VCAM-1 mRNA in endothelial cells. Secretome analysis revealed the ability of PCa TA-NKs to release pro-inflammatory cytokines/chemokines involved in monocyte recruitment and M2-like polarization. Finally, CMs from PCa pTA-NKs recruit THP-1 and peripheral blood CD14^+^ monocyte and polarize THP-1 and peripheral blood CD14^+^ monocyte-derived macrophage towards M2-like/TAM macrophages. Our results show that PCa pTA-NKs acquire properties related to the pro-inflammatory angiogenesis in endothelial cells, recruit monocytes and polarize macrophage to an M2-like type phenotype. Our data provides a rationale for a potential use of pNK profiling in PCa patients.

## Introduction

Prostate carcinoma (PCa) is the one of most commonly diagnosed cancer in males worldwide (1). Surgery and radiation therapy (2) are still important treatment options, as well as chemotherapy (3) and hormonal therapy (4). Recently, immunotherapy came of age as a possible effective strategy for PCa therapy (5). Several immunotherapeutic approaches have been proposed for PCa, that include dendritic-cell based vaccines, whole tumor cell vaccines, vector-based vaccines and antibodies. Currently FDA-approved immunotherapy approaches for PCa include the Sipuleucel−T (a dendritic-cell-based agent) and pembrolizumab (a checkpoint inhibitor that targets the PD-1/PD-L1 axis), while others are in clinical trials.

**Graphical Abstract:**
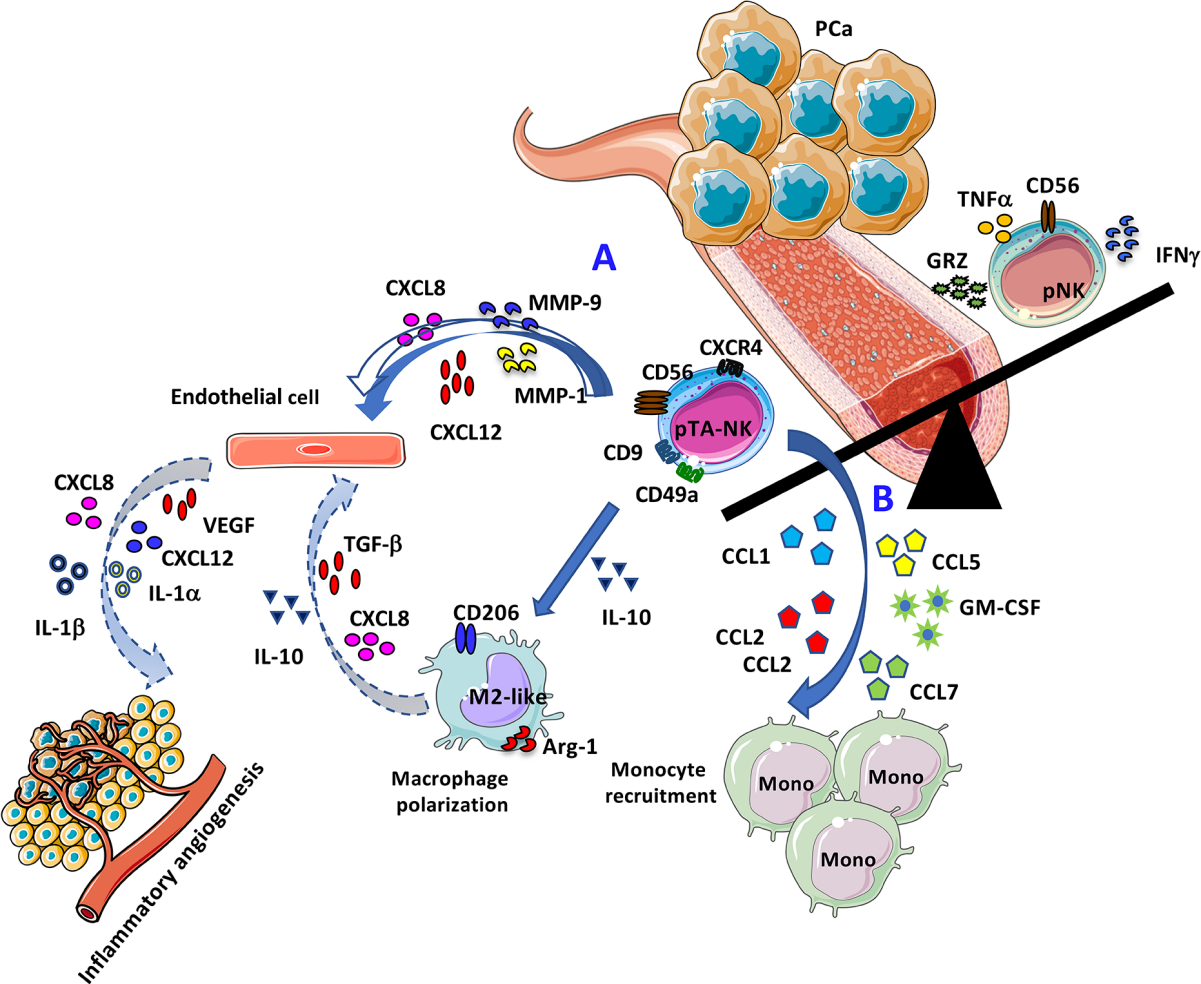
Representative cartoon illustrating the pro-angiogenic features of PCa pTA-NKs. **(A)** Direct effects of PCa pTA-NKs in supporting angiogenesis by interacting with endothelial cells. **(B)** Proposed model for PCa pTA-NK pro-angiogenic activities via monocyte recruitment and macrophage polarization.

Evasion from immune system surveillance and induction of an inflammatory microenvironment are among host-dependent biological features, widely accepted as cancer hallmarks, as defined by Hanahan and Weinberg (6) and which play a role in prostate cancer. Based on their extreme cell plasticity, inflammatory cells from innate and adaptive immunity can acquire tumor-promoting phenotypes and functions in cancer patients. Acquisition of a tolerogenic state, anergy/exhaustion and induction of inflammatory angiogenesis are some of these aberrant functions (7–11).

Natural killer (NK) cells are large granular lymphocytes endowed with an inherent capability to kill virally infected and malignant cells, also participating to the modulation of the immune system, through their production of numerous cytokines and chemokines.

NK cells have been included in the Type-1 Innate Lymphoid cell group (ILC-1), based on their capability to produce IFNγ, following T-bet and EOMES expression by the ID2^+^ ILC precursor (12). A study by the group of Eric Vivier, placed NK cells as cell subset originating from a cell lineage different from ILC-1 (13). While ILC-1 and NK cells share the ability to produce IFNγ in a T-bet dependent manner, NK cells functionally differ from ILC-1 for their cytotoxic abilities, via IFNγ and perforin production (13).

NK cells constitute approximately 5–15% of circulating lymphocytes in healthy adults, representing one of the three major lymphocyte population. Although lymphocytic in origin, NK cells are considered part of the innate immune system, since they do not require antigen presentation for target recognition. They exert effector functions that include cytotoxic activity and cytokine production, during antiviral and antitumor responses (14). Similarly to several immune cells (7–11), NK cells have been described to acquire a tolerogenic behavior and to be altered in their cytotoxic activities in different cancer types (7, 9–11, 15–20). However, pro-inflammatory, pro-tumor NK cells still represent an under investigated cell type; only few studies focused on the ability of polarized NKs to support cancer by acquiring pro-angiogenic phenotypes and functions (7, 10, 15, 16, 18). Major mechanisms associated with impaired NK cell function in cancer patients, are downregulation of lytic perforin and granzyme production, accompanied with reduction of degranulation capabilities, together with reduction of NKG2D (a relevant NK cell activation receptor) expression (21–23). The ligands for NKp30 and NKp46 have been found to be expressed in prostate cancer cell lines, and the blockade of the interaction between the Natural Cytotoxicity Receptors (NCR) with their ligand, can inhibit tumor cell growth (24).

However, studies on prostate cancer associated NK cell phenotype and functions remain limited (20, 25, 26). Isolation of tumour-infiltrating immune components is challenging, due to the small size of prostate biopsies, and the absence of stromal compartments. A study by Daniel Olive laboratory showed that inherent and tumour-driven immune tolerance in the prostate microenvironment impairs NK cell antitumor activity (20). Interestingly, this study also showed enrichment of CD56^bright^ NK cells in tumor tissue, together with impaired NK cell functions, both in tumor tissues and in the peripheral blood (20). Here, we focused on peripheral blood NK cells in PCa patients, with the aim to evaluate their different phenotype and functional profiles in a perspective of a potential liquid biopsy-based procedure.

Two major subsets of NK are mostly present in the peripheral blood (pNK): the cytotoxic CD56^dim^CD16^+^ NK cell subset, (90–95% of pNK) and the low cytotoxic, highly cytokine producing NK cell subset, CD56^bright^CD16^-/low^ (14).

Our research group has identified a new pro-angiogenic NK cell subset in non-small-cell lung carcinoma (NSCLC), described as CD56^bright^CD16^-^VEGF^high^PlGF^high^CXCL-8^+^IFNγ^low^ NK cells (10, 16), supporting a role of NK cells in the inflammatory pro-angiogenic switch in solid tumors. These NK cell population is similar to a peculiar NK subset that has been found within the developing decidua, the decidual NK cell (dNK). dNK cells exhibit the CD56^superbright^CD16^-^CD9^+^CD49a^+^ phenotype and are closely linked with vascularization of the decidua and embryo implantation, in both humans and mice (27, 28). dNK cells produce VEGF, PlGF, and CXCL8, are poorly cytotoxic and are associated with induction of CD4^+^ T regulatory (Treg) cells (27, 28). We characterized pro-angiogenic NK cells also in the peripheral blood (tumour associated NK cells, pTA-NKs) and tissue infiltrate (tumour infiltrating NK cells, TI-NKs) in colorectal cancer patients. These NK cells also display pro-angiogenic features as those in NSCLC patients (16). NK cells in the peripheral blood of NSCLC and CRC, in particular the CD56^bright^CD16^low/−^, share some similarities with the respective TI-NKs, and although they can be defined as decidual NK-like, feature of pregnant women, a similar population is present in both male and female cancer patients (10, 16, 29, 30). We identified TGFβ, a major immunosuppressive cytokine in the tumour microenvironment (TME) (31, 32), as an inducer of the inflammatory/pro-angiogenic switch of cytolytic NK, cells both at tissue and peripheral levels (16). Also, we found that STAT3/STAT5 activation regulates the polarization in CRC NK cells and that STAT5 chemical inhibition, with the anti-psychotic agent Pimozide, interferes with this process (15, 30).

Here, we show, for the first time, that NK cells isolated from peripheral blood of patients with PCa (PCa pTA-NKs), acquire a pro-inflammatory and pro-angiogenic phenotype, characterized by increased expression of the surface antigens CD56, CD9, and CD49a. Analysis of CM of FACS-sorted NK from PCa blood samples, allowed the identification of pNK signatures, characterized by up-regulation of cytokines and chemokines with pro-inflammatory and pro-angiogenic (CXCL8/IL-8) properties, as well as factors involved in the extracellular matrix (ECM) remodeling cascade (MMP-1, MMP-9, uPAR); pro-monocyte recruiting features (CCL1, CCL2, CCL5) and properties involved in M2-like macrophage polarization (IL-10). CM of FACS-sorted pNK cells from PB of PCa patients were able to recruit THP-1 and peripheral blood CD14^+^ monocytes and to polarize THP-1 differentiated macrophages and PB CD14+ place at apex monocyte-derived macrophages towards M2-like/TAM, at transcript level.

Increasing evidence suggests that polarized NK cells are present in the peripheral blood of patients with several types of cancer (9, 10, 15, 16, 29, 30, 33) and their altered profile could be a relevant feature. The idea that NK cells can be envisaged as biomarkers for PCa have been previously explored (26, 34–36). Also, a clinical trial is exploring the significance of circulating NK cells in metastatic PCa (https://clinicaltrials.gov/ct2/show/NCT02963155). Our results provided the characterization of PCa pTA-NK cells, focusing on their polarization state, pro-inflammatory and pro-angiogenic features, for possible NK cell tracing and profiling in PCa patients.

## Materials and Methods

### Sample Selection and Patient Characteristics

Peripheral blood (PB) samples (15–20 ml of whole blood, EDTA) were obtained from patients with prostate adenocarcinomas (ADK, n = 35). Controls (HC, n=27) included peripheral blood of healthy, tumor-free, male individuals. Patients with diabetes, human immunodeficiency virus (HIV)/hepatitis C virus (HCV)/hepatitis B virus (HBV) infection, chronic inflammatory conditions, treated with chemotherapy or radiotherapy, iatrogenically immunosuppressed or subjected to myeloablative therapies, were excluded to the study. The study was approved by the institutional review board ethics committees (protocol no. 0024138 04/07/2011 and protocol no.10 2 10/2011, within the study PROSTATEST) and according to the Helsinki Declaration of 1975 as revised in 2013. All patients enrolled in the study signed the informed consent, in accordance to the Helsinki Declaration of 1975 as revised in 2013. Demographic features of the cohort of PCa patients and controls are showed in **Supplementary Table 1**. Monocytes used for migration studies and monocyte-derive macrophages for polarization experiments were obtained from mononuclear cells from 4 different healthy subjects.

### Cell Culture and Maintenance

The human prostate cancer (PCa) cell lines PC-3, DU-145, LNCaP (all purchased by ATCC) were maintained in RPMI 1640 medium, supplemented with 10% Fetal Bovine Serum (FBS) (Euroclone), 2 mM l-glutamine (Euroclone), 100 U/ml penicillin and 100 μg/ml streptomycin (Euroclone), at 37°C, 5% CO_2_. Cells were routinely screened for eventual mycoplasma contaminations. CM were collected following 72 h of starvation in FBS free RMPI 1640 (Life Technologies), supplemented with 2 mM l-glutamine (Euroclone), 100 U/ml penicillin and 100 μg/ml streptomycin (Euroclone), at 37°C, 5% CO_2_. PCa cell line CMs were used for NK cell polarization as detailed below.

Human umbilical vein endothelial cells (HUVEC, Lonza) were maintained in endothelial cell basal medium (EBM, Lonza) supplemented with endothelial cell growth medium (EGM™ SingleQuots™, Lonza), 10% of FBS, 2 mM l-glutamine (Euroclone), 100 U/ml penicillin and 100 μg/ml streptomycin (Euroclone). HUVECs were used between the three and five passages.

The human monocytic THP-1 cell line (ATCC) was cultured in RPMI 1640 medium, supplemented with 10% FBS, 2 mM l-glutamine (Euroclone), 100 U/ml penicillin and 100 μg/ml streptomycin (Euroclone), at 37°C, 5% CO_2_. Differentiation of adherent THP-1 macrophages was obtained following 48 h of treatments with phorbol-merystate-acetate (5 ng/ml, PMA, Sigma Aldrich) (37).

CD14^+^ monocytes were isolated from PB samples of healthy controls and used as CD14^+^ monocytes or CD14^+^ monocyte-derived macrophages, for cellular and molecular studies. Briefly, total PBMCs were isolated by density gradient stratification with Ficoll Histopaque-1077 (Sigma-Aldrich) and CD14^+^ cells were immediately isolated using the CD14^+^ cell isolation kit (Miltenyi Biotec). CD14^+^ monocyte-derived adherent macrophages were obtained, following CD14^+^ monocyte culture in RPMI 1640 medium, supplemented with 10% Fetal Bovine Serum (FBS), (Euroclone), 2 mM l-glutamine (Euroclone), 100 U/ml penicillin and 100 μg/ml streptomycin (Euroclone), 50 ng/ml M-CSF (Miltenyi Biotec), at 37°C, 5% CO_2_, for 7 days.

### Natural Killer Cell Isolation by FACS Sorting

pNK cells were isolated from peripheral blood mononuclear cells (PBMCs) of PCa ADK and HC subjects. Whole blood was diluted with PBS 1:1 (v/v), then subjected to a density gradient stratification with Ficoll Histopaque-1077 (Sigma-Aldrich), at 500xg for 20 minutes. The white ring interface, composed of total mononuclear cells (MNCs), was collected, washed twice in PBS, then used for subsequent experiments or for pNK isolation. Total MNCs were subject to cell sorting, using a BD FACS-AriaII instrument. Following 30 min of staining with anti-human FITC-conjugated CD45, anti-human PE-conjugated CD14, anti-human PerCP-conjugated CD3 and anti-human APC-conjugated CD56, NK cells was sorted as CD45^+^CD14^-^ CD3^-^CD56^+^ (gating strategy is showed in **Supplementary Figure 1A**). For details of antibodies used, see **Supplementary Table 2**.

FACS-sorted NK cells (2 × 10^5^ cells/ml) were used, following 24 h of culture in serum-free RPMI, for molecular analysis (qPCR) and to collect conditioned media for functional and secretome studies. Following 24 h, supernatants were collected, centrifuged to remove residual dead cells and debris and concentrated using Concentricon (Millipore) with a 3kDa membrane pore cut-off, to obtain concentrated supernatants.

### Cell Treatment with Conditioned Media and Cytokines

For NK cell polarization, total PBMCs (1 × 10^6^ cells/ml) were polarized with 30% of PC-3 or DU-145 or LNCaP CMs (v/v), or TGFβ (10 ng/ml) or IL-6 (25 ng/ml), in RMPI 1640 (Euroclone), supplemented with 10% FBS (Euroclone), 2 mM l-glutamine (Euroclone), 100 U/ml penicillin and 100 μg/ml streptomycin (Euroclone), 100 U/ml IL-2 (R&D), at 37°C, 5% CO_2_, for 72 h. Cells were pulsed with fresh complete RPMI (30%, v/v), alone or with CM or cytokines, at day 0 and at 48 h, during the polarization schedule.

Conditioned media from FACS-sorted NK cells were used to detect the production of pro-inflammatory factors by endothelial cells. 2 × 10^5^ HUVE cells were seeded into six well plates and exposed for 24 h to CM (50 µg/ml of total protein) of PCa pTA-NKs or pNK cell from HC. HUVECs were then harvested and used for real-Time PCR analysis.

THP-1 or peripheral blood CD14^+^ monocytes were used to detect PCa pTA-NK-induced migration, while THP-1 differentiated and peripheral blood CD14^+^ monocyte-derived macrophages were used to investigate pNK-induced polarization, via soluble factors. THP-1 differentiated or CD14^+^ monocyte-derived macrophages were pulsed with CMs (50 µg of total protein) from FACS-sorted pNK cells (either from PCa patients of controls) for 72 h. Cells received CM at day 0 and 48 h of stimulation. Expression of M1-like or M2-like/TAM markers, following polarization, was detected by Real-Time PCR.

### Phenotype Characterization of Conditioned Media-Polarized Peripheral Blood Natural Killer Cells and Prostate Cancer pTA-NKs

The polarization state of either pNK cells exposed to PCa cell line (PC-3, DU-145, LNCaP) conditioned media or pNK cells from PCa patients (PCa pTA-NKs), was assessed by flow cytometry for surface antigen expression. Briefly, 2.5 × 10^5^ of total PBMCs per FACS tube were stained for 30 min at 4°C with anti-human monoclonal antibodies (mAbs) as follows: PerCP-conjugated anti-CD3, APC-conjugated anti-CD56, FITC-conjugated anti-CD16, PE-conjugated anti-CD9, PE-conjugated anti-CD49a, PE- conjugated anti-NKG2D, PE-conjugated anti-PD-1, PE- conjugated anti-TIM-3 (all purchased by Miltenyi Biotec). Following Forward/Side Scatter setting, NK cells were identified as CD3^-^ and CD56^+^ cells (total NK cells). CD16 and NKG2D expression was evaluated on CD3^-^CD56^+^ (total NK) gated cells. Finally, CD56 brightness, the expression of the dNK markers CD9, CD49a, expression of CXCR4 and the expression of the exhaustion markers PD-1 and TIM3, were evaluated on total CD3^-^CD56^+^NK cells. For details on antibodies used, see **Supplementary Table 2**.

### Degranulation Assay

NK cell degranulation ability, as detected by CD107a production, was evaluated both in NK cells from PCa and HC clinical samples or in HC NK cells pre-polarized by 72 h of exposure to PC-3 and DU-145 cell conditioned media. Total MNCs (1 × 10^6^ cells/ml), isolated from PCa patients and controls were cultured, overnight, in RMPI 1640 (Euroclone), supplemented with 10% FBS (Euroclone), 2 mM l-glutamine (Euroclone), 100 U/ml penicillin and 100 μg/ml streptomycin (Euroclone), 100 U/ml IL-2 (R&D), at 37°C, 5% CO_2_.

2 × 10^5^ MNCs were co-incubated with 2 × 10^5^ K562 (E:T ratio of 1:1), in the presence of anti-CD107a- FITC (BD Bioscience) MNCs or K562 alone were used as controls for basal degranulation activities on effector and target cells. Cells were stimulated for 6 h with PMA (10 ng/ml) and ionomycin (500 ng/ml) (both from Sigma), in the presence of GolgiStop plus GolgiPlug (both from BD Biosciences), for 5 h. Finally, the expression of CD107a was detected on CD3^+^CD56^+^ NK cells, by flow cytometry. To determine the degranulation efficiency, the basal levels of NK cell degranulation was subtracted from the NK cells/K562 co-culture.

### Intracellular Staining for Cytokine Detection of Conditioned Media-Polarized Peripheral Blood Natural Killer Cells

For intracellular cytokine detection, 2 × 10^6^ PBMCs from PCa-ADK patients or HC were cultured, overnight, in RPMI 1640 (EuroClone) supplemented with 10% FBS (Life Technologies,) 1% (v/v) l-Glutammine (Sigma), 100 U/ml penicillin, 100 µg/ml streptomycin (Euroclone) and IL-2 (100 U/ml; R&D Systems) at 37°C and 5% CO_2_. For intracellular staining, the third day of polarization, cells were stimulated for 6 h with PMA (10 ng/ml) and ionomycin (500 ng/ml) (both from Sigma), in the presence of GolgiStop plus GolgiPlug (both from BD Biosciences). Cells were collected and stained for NK cell surface markers, as previously described, washed with PBS and treated with Cytofix and Cytoperm fixation and permeabilization kit (BD) for 10 min at 4°C. Cells were then washed in PBS and stained with PE-conjugated anti human CXCL8, CXCL12 (R&D System), IFNγ, TNFα, GranzymeB (Myltenyi Biotec) for 30 min. For indirect staining, cells were incubated for 1 h at 4°C with primary unlabelled antibodies anti-human Angiopoietin 1, anti-human Angiogenin, (all purchased from Abcam), washed and then stained with secondary PE-conjugated antibody anti-mouse IgG, for 30 min, at 4°C. Cytokines production was detected by flow cytometry, using a BD FACS CantoII analyzer. Isotype control and the secondary antibody alone were used as staining controls. For details on antibodies used, see **Supplementary Table 2**.

### Secretome Analysis of Prostate Cancer pTA-NKs

The secretome of conditioned media (50 µg of total protein) of FACS-sorted pNK was assessed, using the Human Angiogenesis Array C1000 (RayBiotech, Inc., Norcross GA) to detect cytokines and chemokines release, as detailed before (30). A pool of three ADK or HCCMs was used. Chemiluminescent signals (revealed as black dots) were captured by membrane exposure to Amersham Hyperfilm. Arrays were computer scanned using the Amersham Imager 680 Analyzer and optical density was determined using the ImageJ software.

### Network Formation Assay on Endothelial Cells

HUVEC cells (1.5 x10^4^ cells/well) were seeded in a 96 well plate, previously coated with 50 µL of 10 mg/ml polymerized Matrigel (BD). After exposure to conditioned media (50 µg/ml total protein, pools of CM from 3 different ADKs or HCs), in serum-free EBM medium, HUVECs were then incubated at 37°C, 5% CO_2_ for 24 h. The formation of capillary-like structures was detected by microphotographs, using an inverted microscope (Zeiss). The number of master segment and master segment length, as indicators of tube formation efficiency, were determined, using ImageJ software and the Angiogenesis Analyzer tool.

### Detection of THP-1 Cell and Peripheral Blood CD14+ Monocyte Recruitment by Prostate Cancer pTA-NKs

Migration assay was performed using modified Boyden chambers. 5 × 10^4^ THP-1 or CD14^+^ monocytes were resuspended in 500 μl of serum-free RPMI and loaded into the upper compartment of the Boyden chamber. The lower chambers were filled with 250 μl of serum-free RMPI medium, supplemented with conditioned media (50 µg/ml total protein, pools of CM from 3 different ADKs or HCs), ADK or HC pNK cells. 5 μm pore-size polycarbonate filters (Whatman, GE Healthcare Europe GmbH, Milan, Italy) previously pre-coated with 2 μg/ml of fibronectin, were used as interface between the two chambers. The Boyden chambers were incubated for 6 h at 37°C. Filters were recovered, cells on the upper surface mechanically removed with a cotton swab. Cells migrated toward the filter surface, were fixed with ethanol at serial percentage (70%, 100%), finally rehydrated in water. Filters were stained with 10 µg/ml DAPI (Vectashield, Vector Laboratories,) and incubated at room temperature, protected from light, for 10 min. Cells in the filters were counted in a double-blind manner in five consecutive fields/filter, with a fluorescent microscope (Nikon Eclipse).

### Quantitative Real-Time PCR

Total RNA was extracted from HUVECs, THP-1 macrophages or peripheral blood CD14^+^ monocyte-derived macrophages, exposed to CM from FACS-sorted PCa pTA-NKs or HC pNK cells, using the small RNA miRNeasy Mini Kit (Thermo Fisher) and quantified by Nanodrop Spectrophotometer. Following genomic DNA removal, by DNase I Amplification Grade (Thermo Fisher) treatment, reverse transcription was performed on 500 ng of total RNA using SuperScript VILO cDNA synthesis kit (Thermo Fisher). Real-time PCR was performed using SYBR Green Master Mix (Thermo Fisher) on QuantStudio 6 Flex Real-Time PCR System Software (Applied Biosystems, Thermo Fisher Scientific, USA). All reactions were performed in triplicate. The GAPDH gene was used as housekeeping and results were showed as 2^^−ΔCt.^ HUVECs or THP-1 macrophages in their respective basal medium alone, were used as baseline controls. Primer sequences are provided in **Supplementary Table 3**.

### Statistical Analysis

Statistical differences between two datasets were determined using two tailed t-test. For multiple datasets, analysis of variance (ANOVA) followed by Tukey’s post-hoc test was used. P values (p) ≤ 0.05 will be considered statistically significant. Data were analysed using the GraphPad Prism8 (San Diego, CA). Flow cytometry data were analysed using the BD FACS-Diva and FlowJo-v10 software.

## Results

### pTA-NKs From Prostate Cancer Patients Exhibit a Pro-inflammatory, Pro-angiogenic, Exhausted Phenotype

**Figure 1:**
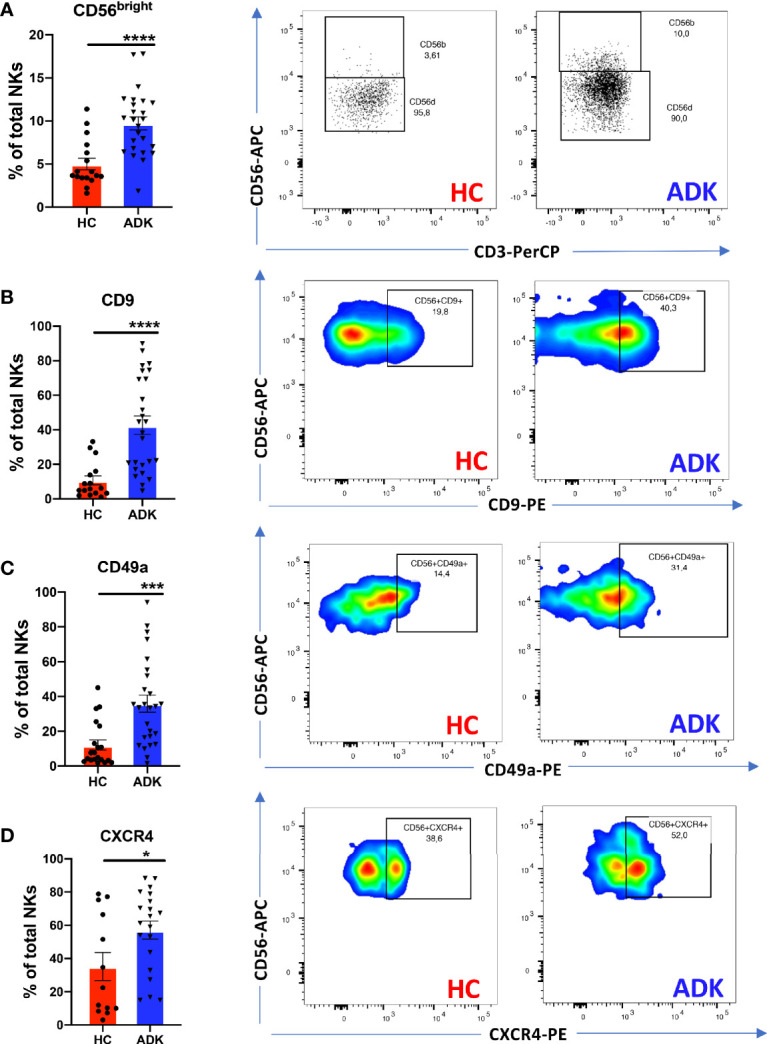
pNK cell polarization in peripheral blood of PCa patients. PCa TA-NKs have increased numbers of CD56^bright^ NKs as compared with those from HC **(A)**. Peripheral blood NK (pTA-NKs) from PCa patients significantly express higher levels of the dNK cell markers CD9 **(B)** CD49a **(C)** and CXCR4 **(D)**, as compared with those from HC. Every dot in dots/bars graph refers to single patients or control. Representative dot plots show the specific antigen expression (as % of total pNK cells). Data are showed as mean ± SEM, t-student test, ^*^p<0.05, ^***^p<0.001, ^****^p<0.0001. HC, healthy controls; ADK, prostate cancer adenocarcinoma.

**Figure 2:**
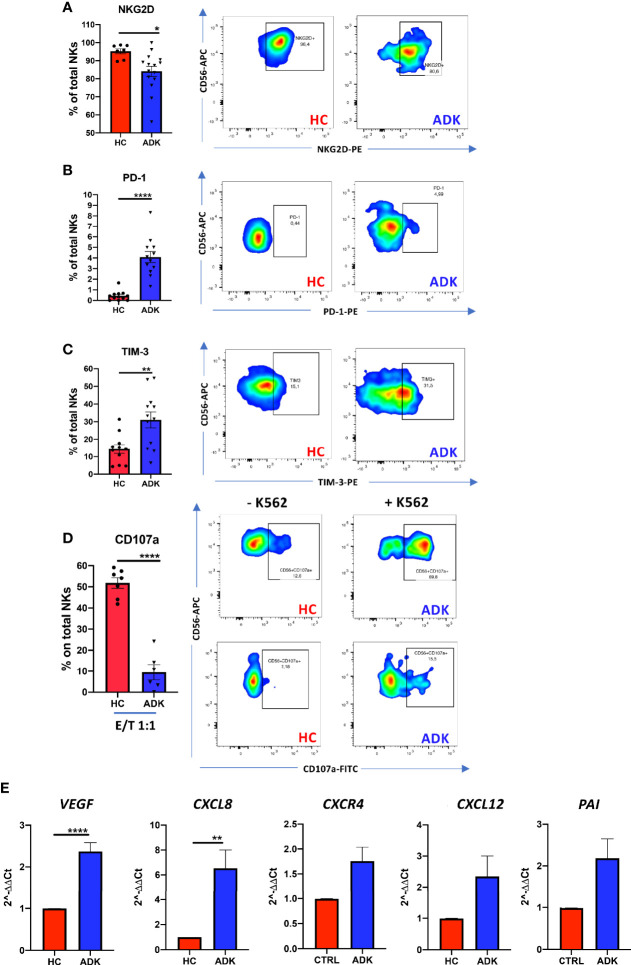
pNK cell exhaustion and degranulation activities in peripheral blood of PCa patients. PCa pTA-NKs have decreased levels of the NKG2D activation markers **(A)**, increased expression of the PD-1 **(B)** and TIM-3 **(C)** exhaustion markers and impaired degranulation abilities against the K562 cells **(D)**. panel D shows NK cell degranulation capabilities, alone or co-incubated with K562 cells in PCa p-TA-NKs and NK cell from healthy controls. Every dot in dots/bars graph refers to single patient or control. Representative dot plots show the specific antigen expression (as % of total pNK cells). pNK cells, FACS-sorted from patients with ADK-PCa have increased expression of the pro-inflammatory factors *VEGF, CXCL8, CXCR4, CXCL12, PAI* **(E)**. qPCR have been performed using pNK cell from 3 PCa patients and 3 controls, in triplicate. Data are showed as mean ± SEM, t-student test, ^*^p<0.05, ^**^p<0.01, ^****^p<0.0001. HC, healthy controls; ADK, prostate cancer adenocarcinoma.

We investigated whether pNK from PCa patients are characterized by a pro-inflammatory and pro-angiogenic phenotype. Flow cytometry analysis of CD56 and CD16 surface antigen expression revealed that the CD56^+^CD16^+^ NK cells are the predominant subset in the peripheral blood in PCa-ADK and HC samples (**Supplementary Figure 1A**). We found increased frequency of CD56^bright^ NK cells in the peripheral blood of patients with PCa ADK (^****^p ≤ 0.0001) (**Figure 1A**). Peripheral blood NK cells from PCa-ADK samples express also higher levels of the decidual-like markers CD9 (**Figure 1B**) (^****^p ≤ 0.0001), CD49a (**Figure 1C**) (^***^p ≤ 0.001), as compared with those isolated from healthy controls. We also found increased expression of CXCR4 on NK cells from PCa-ADK samples (**Figure 1D**) (^*^p ≤ 0.05). We observed that PCa pTA-NKs have reduced expression of the NKG2D activation marker (^*^p ≤ 0.05), together with increased levels of the exhaustion markers PD-1 (^****^p ≤ 0.0001) and TIM-3 (^**^p ≤ 0.01), as compared to those from HC (**Figures 2A–C**). Also, PCa TA-NKs exhibit reduced ability to degranulate against K562 cells (^****^p ≤ 0.0001), as compared to those from HC (**Figure 2D**).

Real-time PCR results showed that TA-NKs cells, FACS-sorted from PCa-ADK, have increased expression of mRNA for the pro-inflammatory factors *CXCL8* (^**^p ≤ 0.01), *CXCL12* and *PAI* and confirmed the increased RNA expression of *CXCR4*, as well as *VEGF* (^****^p ≤ 0.0001), as compared to NK isolated form the peripheral blood of healthy controls (**Figure 2E**).

### pTA-NKs From Prostate Cancer Patients Exhibit a Secretome Profile Enriched in Pro-Inflammatory, Pro-Angiogenic Cytokines, and Chemokines Involved in Monocyte Recruitment and Polarization

**Figure 3:**
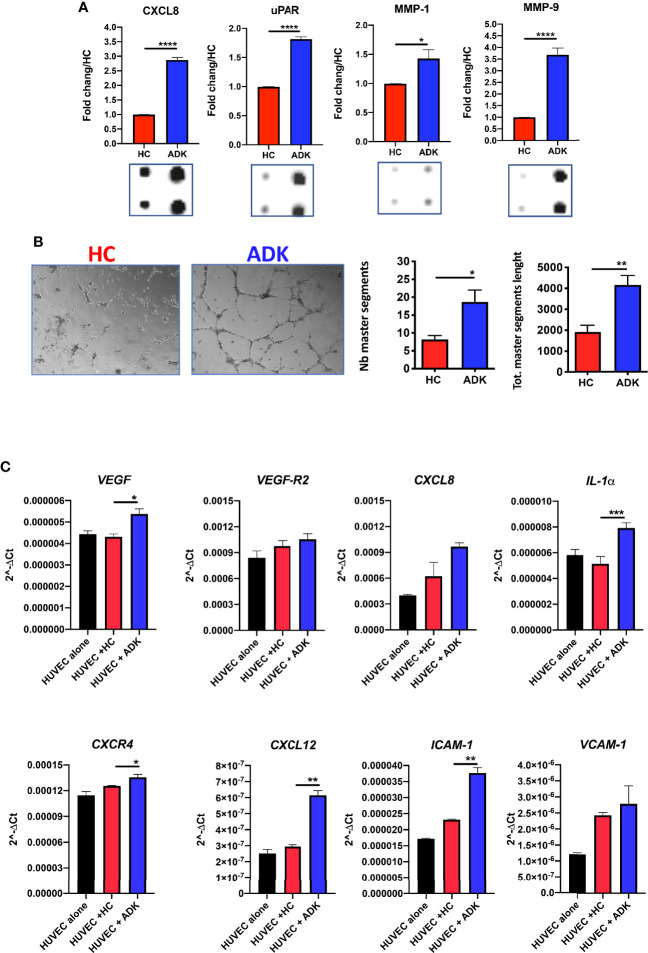
Pro-inflammatory activities of pTA-NKs from PCa patients on endothelial cells. Conditioned media (CM) form FACS-sorted PCa pTA-NKs are enriched in pro-inflammatory and tissue-remodelling factors, such CXCL8, uPAR, MMP-1, MMP-9 **(A)** and functionally support the formation of capillary like structures in human umbilical-vein endothelial cells (HUVEC) on matrigel **(B)**. HUVE cells exposed to conditioned media of PCa pTA-NKs express higher levels of pro-inflammatory factors like *VEGF, VEGF-R2, CXCL8, CXCR4, CXCL12, ICAM-1, VCAM-1, IL1-α*, as compared to those exposed to conditioned media released by healthy control NK cells **(C)**. Capillary like-structure formation and qPCR on HUVECs have been performed using CM of pNK cell from 3 PCa patients and 3 controls, in triplicate, on 4 different HC. Data are showed as 2^^-ΔCt^ values, mean ± SEM, ANOVA, ^*^p<0.05, ^**^p<0.01, ^****^p<0.0001. The condition HUVEC (black bar) stands for HUVE cells alone, as baseline condition. HC, healthy controls; ADK, prostate adenocarcinoma.

**Figure 4:**
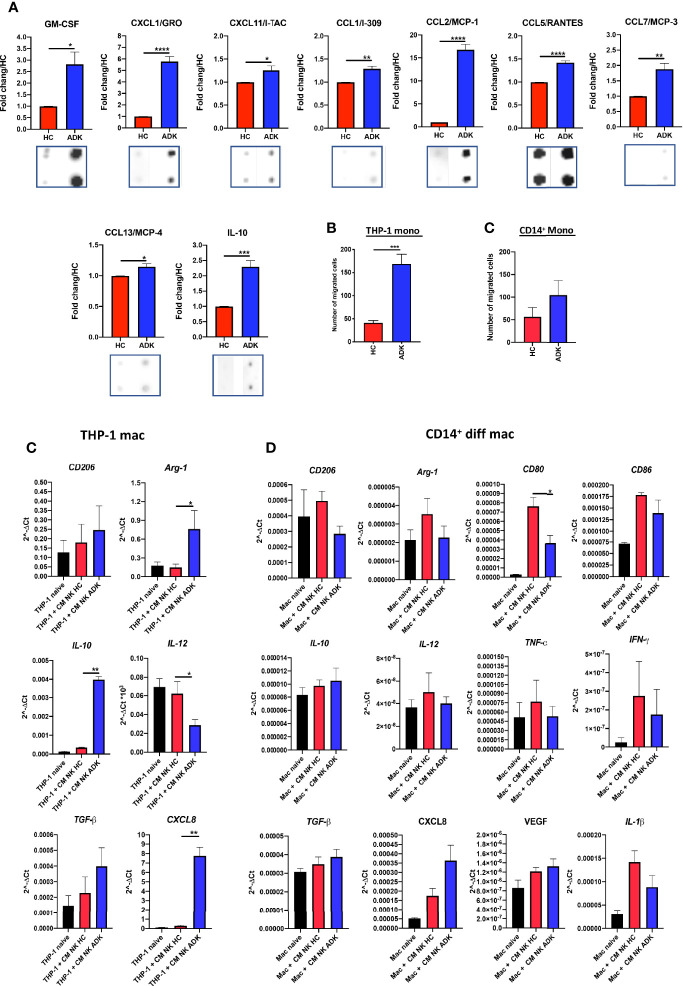
Effects of PCa pTA-NKs on monocyte recruitment and polarization. Conditioned media from PCa pTA-NKs are enriched with factors involved in macrophage recruitment (GM-CSF, CXCL1/GRO, CXCL11/I-TAC, CCL1/I-309, CCL2/MCP-1, CCL5/RANTES, CCL7/MCP-3, CCL13/MCP-4, and polarization (IL-10) **(A)** and can recruit THP-1 and CD14^+^ monocytes as compared with those from heathy controls **(B, C)**, as revealed by the migration assay (Boyden Chambers). Exposure to conditioned media of pNK from PCa patients for 72 h result in THP-1 ability to express higher levels of M2-like/TAM markers (*CD206, Arg-1*, *IL-10, ARG1, CXCL8, TGFβ*) and reduced expression of *IL-12* (M1-like marker) **(C)**. Data from THP-1 were extended, using a larger gene panel *(CD206, Arg-1, CD80, CD86, IL-10, IL-12, TNFα, IFNγ, TGFβ, CXCL8, VEGF, IL-1β*) on peripheral blood CD14^+^ monocyte derived macrophages **(D)**. CMs were pooled from pNK cells FACS sorted from 3 different PCa patients or controls. Arrays were performed in duplicates. q-PCR were performed using CMs pooled from pNK cells FACS sorted from 3 different PCa patients or controls, and used for 4 different experiments (THP-1) and for 4 different donors of peripheral blood CD14^+^ monocyte-derived macrophages, in triplicate. Data are showed as mean ± SEM, ANOVA, ^*^p<0.05, ^**^p<0.01, ^***^p<0.001, ^****^p<0.0001. HC, healthy controls; ADK, prostate adenocarcinoma; naive indicates THP-1 cells in control medium.

To investigate whether the acquisition of the pro-inflammatory phenotype in PCa pTA-NKs would correlate with their capability to release soluble factors involved in direct and indirect induction of inflammatory-angiogenesis, we investigate the contents of CM from PCa TA-NKs. We characterized the production of secreted proteins from PCa TA-NKs using a commercially available angiogenesis-membrane array kit. The overall secretome analysis (**Supplementary Figure 2**) revealed signatures characterizing PCa-ADK pTA-NKs involved in inflammation and angiogenesis (CXCL8) (**Supplementary Figure 2**, **Figure 3A**), tissue remodelling (MMP-1, MMP-9, uPAR) (**Supplementary Figure 2**, **Figure 3A**), monocyte recruitment (CXCL1, CCL2, as the most up-regulated) (**Supplementary Figure 2**, **Figure 4A**) and M2-like macrophage polarization (IL-10) (**Supplementary Figure 2**, **Figure 4A**).

### pTA-NKs From Prostate Cancer Patients Functionally Support Inflammatory Angiogenesis In Vitro

We further investigated whether PCa-ADK pTA-NKs, expressing pro-inflammatory cytokines (also involved in angiogenesis), chemokines and chemokine receptors, were also effectively able to induce network formation in HUVECs *in vitro*. We found that CM of pNK cells isolated PCa-ADK samples have higher contents of the pro-inflammatory and tissue-remodelling factors CXCL8/IL-8 (^****^p≤0.0001), MMP-1 (^*^p≤0.05), MMP-9 (^****^p≤0.0001), uPAR (^****^p≤0.0001) (**Figure 3A**, **Supplementary Figure 2**). To detect whether conditioned media of inflammatory NK cells isolated PCa-ADK samples were effectively able to induce network formation in HUVE cells, we treated HUVE cells with these conditioned media. We found that conditioned media of pNK cells isolated from PCa-ADK are able to induce the formation of capillary-like structures by HUVE cells, on a matrigel layer (^*^p ≤ 0.05; ^**^p ≤ 0.01), as a consequence of their pro-inflammatory secretome (**Figure 3B**). Real-time PCR results showed that HUVE cells, exposed for 24 h to CM from PCa-ADK pTA-NKs have a pro-inflammatory phenotype with increased mRNA expression of *VEGF* (^*^p ≤ 0.05), *VEGF-R2*, *CXCL8* and of factors involved in vascular inflammation and immune cells mobilization, such as *CXCR4* (^*^p ≤ 0.05), *CXCL12* (^**^p ≤ 0.01), *ICAM-1* (^**^p ≤ 0.01), *VCAM-1*, together with induction of *IL-1*α (^***^p ≤ 0.001) (**Figure 3C**).

### pTA-NKs From Prostate Cancer Patients Can Recruit Monocytes and Induce an M2-Like/TAM Features In Vitro

**Figure 5:**
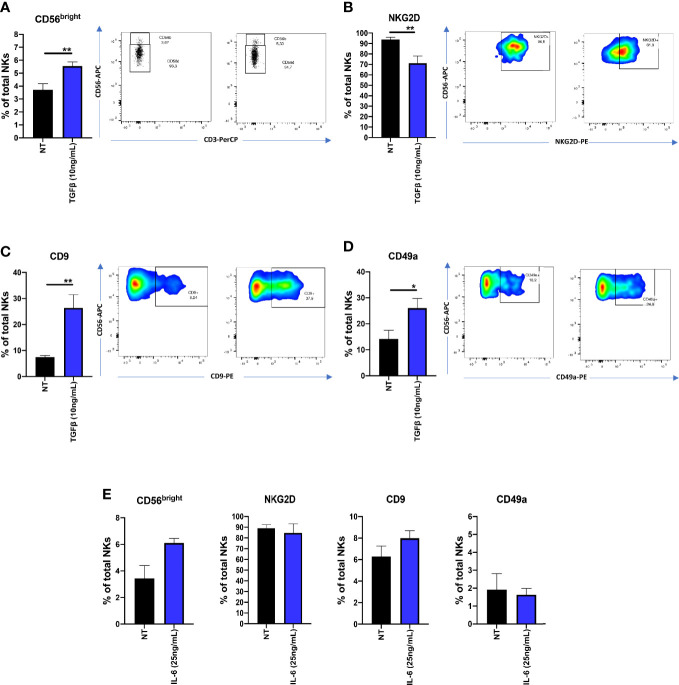
Effects of TGFβ and IL-6 on NK cell polarization. NK cells from healthy donors, following 72 h of exposure to TGFβ (10 ng/ml) or IL-6 (25 ng/ml) were analyzed for their polarization state, by multicolor flow cytometry. TGFβ induced the CD56^bright^CD9^+^CD49a^+^NKG2D^low^ phenotype in cytolytic NKs **(A–D)**. The same effect was not observed on cells polarized with IL-6 **(E)**. Polarization experiments were performed on seven different healthy donors for TGFβ and four different healthy donors for IL-6. Data are shown as mean ± SEM, ANOVA, ^*^p<0.05, ^**^p<0.0. NT, not-treated cells.

Secretome analysis revealed that conditioned media of pNK cells isolated PCa-ADK samples are enriched in soluble factors involved in macrophage recruitment and polarization (**Supplementary Figure 2**, **Figure 4A**) such as GM-CSF (^*^p ≤ 0.05), CXCL1/GRO (^****^p ≤ 0.0001), CXCL11/I-TAC (^*^p ≤ 0.05), CCL1/I-309 (^**^p ≤ 0.01), CCL2/MCP-1 (^****^p ≤ 0.0001), CCL5/RANTES (^****^p ≤ 0.0001), CCL7/MCP-3 (^**^p ≤ 0.01), CCL13/MCP-4 (^*^p ≤ 0.05) and IL-10 (^***^p ≤ 0.001) (**Figure 5A**). Based on these results, we functionally investigated PCa-ADK NK cells ability to recruit THP-1 or CD14^+^ monocytes, via soluble factors. We observed that CM from PCa pTA-NK cells, FACS sorted from the peripheral blood of PCa-ADK patients, promote the recruitment of THP-1 monocytes (^***^p ≤ 0.001) as compared to CM of NK cells isolated from healthy controls (**Figure 4B**). A similar trend was observed in peripheral blood CD14^+^ monocytes, exposed to CM from PCa pTA-NK as compared to CM of NK cells isolated from healthy controls (**Figure 4C**). We also observed that THP-1 differentiated macrophages, following 72 h of exposure to PCa pTA-NK CMs, displayed increased expression of the M2-like/TAM factors, such as CD206/Mannose receptor, Arg1 (^*^p ≤ 0.05), IL-10 (^**^p ≤ 0.01), TGFβ, CXCL8 (^***^p ≤ 0.001) and decreased expression of the M1-like cytokine IL-12 (^*^p ≤ 0.05) (**Figure 4C**). We extended this analysis on peripheral blood CD14^+^ monocyte-derived macrophages, using a larger gene candidate panel, exposed for 72 h to CMs from FACS-sorted PCa pTA-NK or those from HC (**Figure 4D**). While data on CD206 and Arg1 expression seems to not reflect those observed in THP-1 macrophages, we observed decreased levels of CD80 (^*^p ≤ 0.05) and CD86 (M1-like markers). Results on IL-10 (M2-like) and IL-12 (M1-like), TGFβ and CXCL8 (both M2-like), show a trend similar to that observed in THP-1 macrophages (**Figure 4D**). In addition, we found that peripheral blood CD14^+^ monocyte-derived macrophages, exposed for 72 h to CMs from FACS-sorted PCa pTA-NK or those from HC, have a trend in increased VEGF (M2-like) transcript, together with decreased expression of TNFα, IFNγ and IL-1β pro-inflammatory (M1-like) cytokines (**Figure 4D**).

### TGFβ and IL-6 Effects on Natural Killer Cell Polarization

Since TGFβ and IL-6 have been found to be abundant in serum and plasma levels of PCa patients, we investigated TGFβ and IL-6 abilities to polarized cytolytic NK cells from heathy donors. We found that, following 72 h of TGFβ (10 ng/ml) exposure, NK cells increase the surface expression of CD56 (^**^p ≤ 0.01) (**Figure 5A**), decreased NKG2D expression (^**^p ≤ 0.01) (**Figure 5B**) and increased surface expression of CD9 (^**^p ≤ 0.01) and CD49a (^*^p ≤ 0.05) (**Figure 5C-D**). In contrast, IL-6 (25ng/ml), was not able to induce a similar effect (**Figure 5E**).

### Prostate Cancer Cell Lines Conditioned Media Polarize pNK Cells Toward Pro-Inflammatory Angiogenic, Exhausted Natural Killer Cells

**Figure 6:**
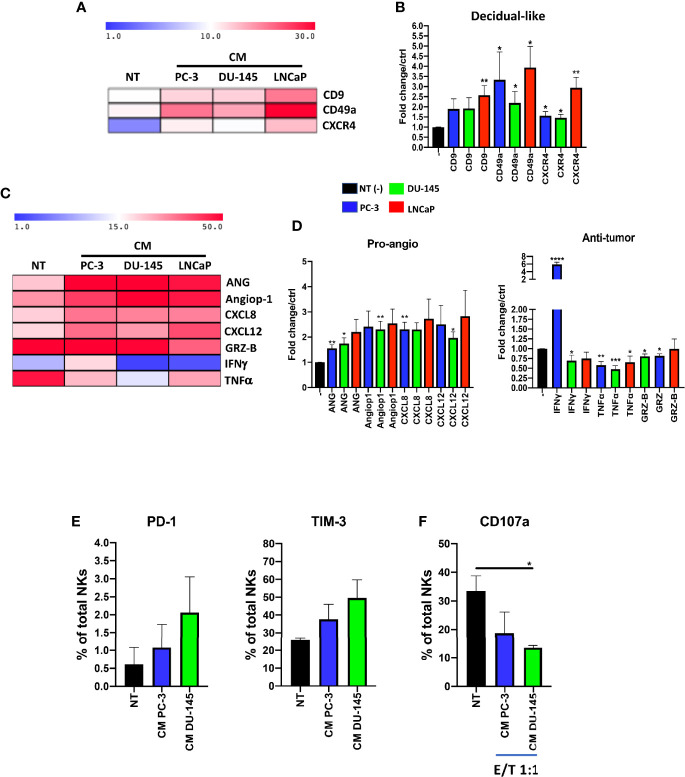
Effects of prostate cancer cell line conditioned media (CM) on pNK cell polarization and functions. NK cells from healthy donors, following 72 h of exposure to conditioned media (CM) from PC-3, DU-145 and LNCaP PCa cell lines, exhibit a pro-inflammatory angiogenic decidual-like phenotype, as revealed by the increased levels of the dNK-like markers CD9, CD49a, CXCR4 **(A, B)**, enhanced production of pro-inflammatory factors (angiogenin, ANG; angiopoietin-1, Angiop1; CXCL8) and reduced production of cytolytic factors (granzyme B, GRZ-B; TNFα; IFNγ) **(C, D)**, as revealed by flow cytometry analysis. Experiments were performed using peripheral blood samples of 5-to-9 independent healthy donors. NK cells from healthy donors, following 72 h of exposure to conditioned media (CM) from PC-3, DU-145 cell lines increase the expression of PD-1 and TIM-3 exhaustion markers **(E)**, together with decreased degranulation capabilities against K562 cells **(F)**. Experiments were performed using peripheral blood samples of 3 independent healthy donors. Data are shown as mean ± SEM, ANOVA, ^*^p<0.05, ^**^p<0.01, ^***^p<0.001. CM, conditioned media/conditioned media from 72 h of SFM PCa cell lines.

To verify the results obtained from PCa pTA-NKs, we used an *in vitro* model mimicking the interaction of the secretome of PCa cells with normal PBMC of healthy donors. Mononuclear cells from peripheral blood were exposed to soluble factors (CM) collected from three different PCa cell lines (PC-3, DU145, LNCaP) and assessed for their expression of decidual and pro-inflammatory, pro-angiogenic markers and polarization state. We found that pNK cells from healthy controls, following 72 hs of exposure to the CM of three different PCa cell lines (PC-3, DU-145, LNCaP) showed increase expression of the CD9, CD49a of CXCR4 (^*^p ≤ 0.05, ^**^p ≤ 0.01) (**Figures 6A, B**). We also found that 72 h of stimulation with CM from the three PCa cell lines resulted in pNK enhanced ability to produce pro-inflammatory and pro-angiogenic factors, such as Angiogenin, Angiopoietin-1, CXCL8 (^**^p ≤ 0.01) and CXCL12 (^*^p ≤ 0.05, ^**^p ≤ 0.01), and decreased ability to produce IFNγ, TNFα and Granzyme-B (^*^p ≤ 0.05, ^**^p ≤ 0.01, ^***^p ≤ 0.001), as detected by flow cytometry (**Figures 6C, D**). Finally, we observed that pNK cells from healthy controls, following 72 h of exposure to the CM of PC-3 and DU-145 cell lines are exhausted, as revealed by the trend of increased levels of PD-1 and TIM-3, together with decreased degranulation capabilities against K562 cells (^*^p ≤ 0.05) (**Figures 6E, F**).

## Discussion

Although immunotherapy has emerged as the “next generation” cancer treatment (38), it is not always successful in the treatment of patients with PCa, for whom the preferential therapeutic options still remain radiotherapy, chemotherapy and androgen deprivation therapy (3, 39–41). This clearly suggests that, to address more efficient immune therapeutic approaches against PCa, a better understanding of how the PCa is able to subvert the host immune system, still remains a major issue and a clinical unmet need. Preclinical and clinical evidences suggest that chronic inflammation plays a crucial role in multiple stages of prostate cancer development (42–44).

The polarization of the immune inflammatory cells in peripheral blood is directed by specific chemokines and cytokines that can shape their state and make them acquire altered phenotype and functions, depending on tumour scenario (7, 10, 11, 45–47).

NK cells have been found to be compromised in several cancers (7, 10, 11, 14–17, 19, 22, 23, 29, 30, 48). Skewed NK cell contribution to tumour progression goes beyond tumour escape and immunosuppression (7, 10, 15, 16, 29, 30). We demonstrated that NK cells in NSCLC cancer (16), colorectal cancer (7) and in malignant pleural effusions (29), acquire pro-angiogenic, pro-inflammatory phenotype and functions, identified as CD56^bright^CD16^−^VEGF^high^CXCL8^+^IFNγ^low^ and share several features and behaviours with the highly pro-angiogenic decidual NK (dNK) cells. This was observed also by other groups in breast and colon cancers (49).

NK cell scenario in PCa is less investigated. Here, we characterized pNK cells isolated from the PB of patients with PCa, in the framework of approved clinical protocols. We found that PB NK cells from PCa patients (PCa pTA-NKs) show a pro-inflammatory and pro-angiogenic polarization, by acquiring the CD56^bright^CD9^+^CD49a^+^CXCR4^+^ phenotype. Our results on increased CD56^bright^ frequency are in line with those showed by Pasero et al. in prostate cancer tissues (20). Similar to these results, we observed that PCa pTA-NKs, have impaired degranulation capabilities. In addition, we also found that PCa pTA-NKs exhibit down-regulation of NKG2D and increased markers of exhaustion, such as PD-1 and TIM-3, as compared with those from healthy controls. Increased expression of PD-1 and TIM-3 have been reported as markers of NK cell exhaustion also in other cancer types (50–55). We found that circulating PCa pTA-NKs were able to express larger amount of the pro-inflammatory and pro-angiogenic factors VEGF, CXCL8, CXCL12, PAI, as compared to those from controls, introducing a new scenario for the possible pro-inflammatory and pro-angiogenic activities of circulating NK cell in PCa.

Based on these first results, we investigated whether soluble-related factors, released by PCa pTA-NKs, might support pro-inflammatory and pro-angiogenic-like behaviour, acting on endothelial cells and cellular components of the innate immune system, such as monocyte or macrophages. NK cells can interact with most of the innate and adaptive cellular components of the immune system (7, 11, 15, 56, 57). Monocytes are the second most represented phagocytes in circulation and in established progressing tumours, were they display an M2-like/TAM phenotype (58–60). M2-like macrophages, induced *in vitro*, have been shown to decrease the susceptibility of tumour cells to NK cell cytotoxicity, with increased PD-L1 and decreased NKG2D ligands in castration-resistant prostate cancer cells (61).

Here, we analysed the PCa pTA-NK production of pro-inflammatory/pro-angiogenic factors, using commercially available protein membrane arrays. We found elevated release of CXCL8/IL-8 by PCa pTA-NK, which can be responsible for the PCa pTA-NK soluble-factor mediated induction of HUVEC capillary-like structures on matrigel. These results support the hypothesis that PCa pTA-NK can potentially promote inflammatory angiogenesis. A number of studies have linked higher serum levels or expression of CXCL8/IL-8 with aggressive prostate cancer. Elevated CXCL8/IL-8 has been reported to correlate with high Gleason score and with AR loss in metastatic disease (62–65). Interestingly, CXCL8/IL-8 was the most abundant factor that we found to be released by PCa pTA-NK.

We also found that PCa pTA-NK can produce factors involved in tissue remodelling and metastasis, such as MMP-1, MMP-9, uPAR. Other studies reported that MMP-1, MMP-9, uPAR play important roles in tissue remodelling with prognostic implication in PCa (66–68). In previously published results, we have shown that MMP-9 is upregulated in peripheral blood NK cells of colon cancer patients and the TIMP-1/MMP-9 axis, as well as uPAR, are altered, as compared to normal circulating NK cells (30).

The crosstalk between NK cells and M1 macrophages plays a crucial role in the protection against infections and tumour development (69–71). In hepatocellular carcinoma (HCC), tumour-derived monocytes have been found to induce dysfunctions in NK cells that were impaired in their ability to produce TNFα and IFNγ (71). CM of M2 type macrophages have been found to decreases the susceptibility of tumour cells to NK cell cytotoxicity, as a result of increased PD-L1 and decreased NKG2D ligands in prostate cancer cells. This has been reported to be mediated through the IL-6 and STAT3 pathway (61).

While macrophage-NK cell crosstalk has been investigated in different cancers (69, 70, 72–74), less studies have investigated the crosstalk in the opposite direction. We assessed the ability of PCa TA-NKs to recruit THP-1 and CD14^+^ monocytes *in vitro*. We found that PCa pTA-NKs have increased ability to stimulate migration of THP-1 and CD14^+^ monocytes, as compared to pNK cells from healthy controls. We also tested whether the PCa pTA-NK released products may impact on macrophage polarization state. We found that THP-1-differentiated and peripheral blood CD14^+^ monocyte-derived macrophages, exposed for 72 h to conditioned media from PCa pTA-NK cells, acquire increase the expression of M2-like/TAM genes (C*D206, ARG-1, IL10, TGFβ*, *CXCL8, VEGF)*, while decreasing the expression of M1-like factors *CD81, CD86*, *IL-12, TNFγ, IFNγ*. These results provide the rational to propose that pro-inflammatory, pro-angiogenic activities by PCa pTA-NKs may also act by shaping monocyte and macrophage polarization and functions. M2-like macrophages/TAMs have been associated with increased tumour angiogenesis and poorer survival in PCa patients (75–77).

IL-2 priming of NK cells from patients with PCa, has been reported to result in distinct NK cell phenotypes and correlates with different NK cytotoxic activities (48). Once again, these cited results, together with our study, point out the important role of the phenotype and functions of NK cells in PCa patients, that could be used, in the future, for immune-profiling of NK cells in PCa.

Based on our previous studies (16, 30), we tested, *in vitro*, the possible contribution of major cytokines, TGFβ and IL-6 in supporting the pro-inflammatory and pro-angiogenic polarization of NK cells from healthy controls.

TGFβ has been largely reported as an inducer of immunosuppression and immune cell escape in diverse cancers (31), including PCa (78). TGFβ is largely present in plasma/serum samples of PCa patients (78). We found that TGFβ induced the CD56^bright^CD9^+^CD49a^+^NKG2D^low^ phenotype in healthy donor derived NK cells.

IL-6 has been reported to be produced by several cancer types, including PCa (79), endowed with pleiotropic effects (80). IL-6 has been reported to impair NK cell functions by activating the STAT3 pathway (80–82). Also, inhibition of IL-6-JAK/STAT3 signalling result in the enhancement of NK cell-mediated cytotoxicity via alteration of PD-L1/NKG2D ligand levels, in castration-resistant prostate cancer cells (82). In our study, we found that IL-6 was not able to induce the CD56^bright^CD9^+^CD49a^+^NKG2D^low^ phenotype in healthy donor derived NK cells.

We validated our findings from clinical samples using an *in vitro* model, mimicking the interaction of PCa soluble factors on cytolytic NK cells, by exposing NK cells from healthy donors to conditioned media of different PCa cell lines (PC-3, DU-145, LNCaP). Pasero et al previously showed that the PC-3 cell line can alter the expression of activation receptors in NK, such as NKG2D, DNAM-1, NKp46, NKp30, together with decreased degranulation capabilities (20). Using three different PCa cell lines, PC-3, DU-145 and LNCaP, respectively, we observed that their conditioned media were able to induce the CD56^bright^CD9^+^CD49a^+^CXCR4^+^ phenotype in NK cells derived from healthy controls, together to the capability to produce Angiogenin, Angiopoietin-1, CXCL8, CXCL12. We confirmed that CM from PCa cell lines induce anergy in healthy donor derived NK cells, with reduced capability to produce the anti-tumour cytokines IFNγ, TNFα and the cytotoxic factor granzyme-B. As a further proof anergy, PC-3 and DU-145 CM-polarized NK cells increase the expression of the PD-1 and TIM-3 exhaustion markers and exhibit reduced degranulation activities.

## Conclusions

Limited data are available of PCa pTA-NKs. A pivotal study by Pasero et al. showed enrichment of CD56^bright^ NK cells in tumour tissue, together with impaired NK cell functions both in tumour tissues and in the peripheral blood (20). Here, we focused on the characterization and phenotyping of peripheral blood NK cells from PCa patients, with the aim to evaluate their different phenotype and functional profiles, as compared to those from heathy controls.

We show that PCa pTA-NKs have a pro-inflammatory and pro-angiogenic phenotype, are endowed with the ability to support angiogenesis, *in vitro*, stimulating endothelial cell activation and functions, are able to recruit monocytes and polarize macrophages via soluble factors. Since obtaining PB NK cells is a relatively easy and poorly invasive procedure, our data provide a rationale for the future use of the pNK profiling in PCa patients to monitor NK cell polarization state and for designing approaches to restore pNK lytic activity in PCa patients.

## Data Availability Statement

The raw data supporting the conclusions of this article will be made available by the authors, without undue reservation.

## Ethics Statement

The studies involving human participants were reviewed and approved by the ethics committees of the University of Insubria (protocol no. 0024138 04/07/2011), Viale Borri, 57-21100 Varese (VA), Varese, Italy, ethics committees IRCCS MultiMedica (protocol no 10 2 10/2011), Via Milanese 300-20099, Sesto San Giovanni (Milan), Milan, Italy. The patients/participants provided their written informed consent to participate in this study.

## Author Contributions

MG, DB, LM, ABo, GB, and ABr: performed the experiments in a previous submission. MG, LM, GB, and ABr performed the experiments in this revision. DB and ABr analyzed the data, performed the statistical analysis, and prepared the figures. FD, PC, AN, and AG provided and collected the clinical samples and provided the clinical support. DB, LM, DN, AA, and ABr conceived the experiments and analyzed the data. DB, LM, DN, AA, and ABr wrote the manuscript. DN and ABr provided funds. All authors contributed to the article and approved the submitted version.

## Funding

This work was supported by the Italian Association for Cancer research (AIRC) within the MFAG 2019-ID 22818, to ABR, the University of Insubria intramural grant, Fondo di Ateneo per la Ricerca FAR 2018 and FAR 2019 to LM and DN, the Italian Ministry of University and Research PRIN 2017 grant 2017NTK4HY, to DN and the Italian Ministry of Health Ricerca Corrente-IRCCS MultiMedica, to AA, DN and ABr. MG is a participant to PhD course in Life Sciences and Biotechnology at the University of Insubria; DB is funded by an “assegno di ricerca,” MIUR.

## Conflict of Interest

The authors declare that the research was conducted in the absence of any commercial or financial relationships that could be construed as a potential conflict of interest.

## Supplementary Material

Supplementary Figure 1 Gating strategy and expression of CD16 in PCa pTA-NKs and controls. **(A)** Gating strategy used for NK cell FACS sorting. **(B)** pTA-NKs from PCa patients share similar pNK cell subset frequency (CD56^+^CD16^+^ and CD56^+^CD16^-^ cells) as those from peripheral blood samples of healthy controls **(A)**. Data are showed as mean ± SEM, t-test student, **p<0.01. HC: healthy controls; ADK: prostate adenocarcinoma.

Supplementary Figure 2 Secretome profiling of PCa pTA-NKs. Secretome profiling, using an antibody membrane array showed that conditioned media from pNK cells of PCa patients are enriched in several factors directly and indirectly associated with induction of angiogenesis, immunosuppression, M2-like polarization, macrophage recruitment, ECM/tissue remodelling. **(A)** Representative heatmap; **(B)** bar histogram showing the fold change for every modulated factor PCa over HC; **(C, D)** scan acquisition for the angiogenesis antibody-array (C1 and C2), showing overall dots, following exposure to conditioned media (CM) from FACS sorted pNK cells of PCa-ADK patients and controls. Green tables, to identify dots position for array C1 and C2, is provided. CMs were pooled from pNK cells sorted from 3 different PCa patients or HC. Arrays were performed in duplicates. Data are showed as mean ± SEM, t-test student, *p<0.05, **p<0.01, ***p<0.001, ****p<0.0001. ADK, prostate adenocarcinoma; HC, healthy controls.

Supplementary Table 1 Demographic and clinical features of our cohort of PCa patients and controls. Table summarizing the features of our cohorts of patients, with relative sample size. Average of age is showed as mean ± SD. N: sample size, ADK, prostate adenocarcinoma; HC, healthy controls.

Supplementary Table 2 Antibodies used in flow cytometry experiments. The table summarizes the antibodies (primary conjugated, primary not-conjugated, secondary conjugated) used in flow cytometry analysis.

Supplementary Table 3 Primer sequences for oligos used for Real-time PCR. Sequences for forward and reverse oligos used for real-time PCR are showed.

## Abbreviations

ADCC, antibody-dependent cellular cytotoxicity; ADK, adenocarcinoma; ANG, angiogenin; ANGIOP1, angiopoietin 1; ANOVA, analysis of variance; CCL, chemokine ligand (C-C motif); cDNA, complementary DNA; CM, conditioned media; CRPC, castration-resistant prostate cancer; CXCL, chemokine Ligand (C-X-C motif); DNAM-1, DNAX accessory molecule 1; dNK cells, decidual Natural Killer cells; EBM, endothelial cell basal medium; EGM, endothelial cell growth medium; FACS, fluorescence-activated cell sorting; FBS, fetal bovine serum; FC, flow cytometry; FSC, forward scatter; GAPDH, glyceraldehyde-3-phosphate dehydrogenase; GM-CSF, granulocyte-macrophage colony-stimulating factor; HBV, hepatitis B virus; HC, healthy control; HCC, hepato cellular carcinoma; HCV, hepatitis C virus; HIV, human immunodeficiency virus; HUVEC, human umbilical vein endothelial cell; ICAM, inter cellular adhesion molecule-1; IFN- γ, interferon γ; IHC, immuno histo chemistry; IL-, interleukin-; I-TAC, interferon-inducible T-cell alpha chemoattractant; mAbs, monoclonal antibodies; MCP-1/CCL2, monocyte chemoattractant protein-1; MMPs, matrix metallo proteinases; MNCs, mono nuclear cells; NK, natural killer; NKG2D, natural killer receptor group 2 D; NSCLC, non-small cell lung cancer; PAI, plasminogen activator inhibitor; PB, peripheral blood cells; PBMCs, peripheral blood mononuclear cells; PCa, prostate cancer; PD-L1, programmed death receptor ligand 1; PGE2, prostaglandin E2; PlGF, placental growth factor; PMA, phorbol merystate acetate; P/S, penicillin/streptomycin; PTA-NKs, prostate tumor-associated natural killer cells; RANTES, regulated upon activation, normal t cell expressed and presumably secreted; SSC, side scatter; STAT, signal transducer and activator of transcription; TAMs, tumor-associated macrophages; TGFβ, transforming growth factor-β; TIMP, tissue inhibitor of metallo-proteinase; TME, tumor microenvironment; TNF α, tumor necrosis factor-α; Treg, T regulatory cells; uPAR, urokinase-type plasminogen activator receptor; VCAM, vascular cellular adhesion molecule-1; VEGF, vascular endothelial growth factor; VEGFR, vascular endothelial growth factor receptor.
