# Supplementary material for: Prostate Cancer Peripheral Blood NK Cells Show Enhanced CD9, CD49a, CXCR4, CXCL8, MMP-9 Production and Secrete Monocyte-Recruiting and Polarizing Factors
Source: Front Immunol. 2021 Jan 25;11:586126. doi: 10.3389/fimmu.2020.586126 (PMC7868409; doi:10.3389/fimmu.2020.586126)
Supplement: Supplementary Figure 1 — Gating strategy and expression of CD16 in PCa pTA-NKs and controls. (A) Gating strategy used for NK cell FACS sorting. (B) pTA-NKs from PCa patients share similar pNK cell subset frequency (CD56+CD16+ and CD56+CD16- cells) as those from peripheral blood samples of healthy controls (A). Data are showed as mean ± SEM, t-test student, **p<0.01. HC: healthy controls; ADK: prostate adenocarcinoma. [file DataSheet_1.docx]

Supplementary Material

**Supplementary Figure 1**

**Supplementary Figure 2**

**Supplementary Table 1**

| **DIAGNOSIS** | **ADK** | **HC (Ctrl)** |
| --- | --- | --- |
| N° of subjects | 35 | 27 |
| Age (mean ± sd) | 70±6.5 | 57±10.16 |

| **ID** | **AGE** | **HISTOLOGY** | **TMN** | **PSA** | **GLEASON** |
| --- | --- | --- | --- | --- | --- |
| V1 | 69 | 68 | pT3b R1 pN0 | 11,4 | 4+5 |
| V2 | 68 | ADK | pT2c pN0 M0 | 9,2 | 4+3 |
| V3 | 74 | ADK | pT2c pN0 M0 | 3,4 | 4+4 |
| V4 | 64 | ADK | pT2c pNx M0 | 2,3 | 3+4 |
| V5 | 74 | ADK | ND | ND | 3+4 |
| V6 | 64 | ADK | ND | ND | 3+4 |
| V7 | 76 | ADK | pT2R1 pN0 M0 | 7,8 | 3+4 |
| V8 | 70 | ADK | pT2R1 pN0 M0 | 12,1 | 4+3 |
| V9 | 53 | ADK | pT2c pN0 M0 | 5,5 | 3+4 |
| V10 | 75 | ADK | cT2a pN0 M0 | 6 | ND |
| V11 | 58 | ADK | Ct2b N0 M0 | 5 | ND |
| V12 | 64 | ADK | pT2a pN0 M0 | 3 | 3+4 |
| V13 | 77 | ADK | cT2b N0 M0 | 5,4 | ND |
| V14 | 75 | ADK | cT1c N0 M0 | 2,7 | ND |
| V15 | 75 | ADK | Ct2b N0 M0 | 7,3 | ND |
| V16 | 76 | ADK | Ct2b N0 M1 | 18 | ND |
| V17 | 75 | ADK | cT3a N0 M0 | 12 | ND |
| V18 | 66 | ADK | pT2c pN0 M0 | 5,5 | 3+3 |
| V19 | 52 | ADK | pT3b pN0 M1 | 68 | 4+4/4+3 |
| V20 | 74 | ADK | cT1c N0 M0 | 4,7 | ND |
| V21 | 77 | ADK | pT2c pN0 M0 | 15 | 3+3 |
| SG1 | 66 | ADK | T3bN1 | 21 | 4+5 |
| SG2 | 70 | ADK | T2Nx | 3 | 3+3 |
| SG3 | 65 | ADK | T2N0 | 8,6 | 3+3 |
| SG4 | 73 | ADK | T3aN1 | 14 | 4+5 |
| SG5 | 72 | ADK | T2N0 | 5,9 | 3+4 |
| SG6 | 66 | ADK | T3N0 | 9,6 | 3+4 (+5) |
| SG7 | 75 | ADK | T2N0 | 5 | 3+4 |
| SG8 | 75 | ADK | T3N0 | 35 | 4+5 |
| SG9 | 67 | ADK | T2N0 | 5.18 | 4+3 |
| SG10 | 78 | ADK | T3aN0 | 8.44 | 3+3 |
| SG11 | 73 | ADK | T3bN0 | 10.1 | 4+3 |
| SG12 | 70 | ADK | T2N0 | 7.5 | 3+4 |
| SG13 | 64 | ADK | T2Nx | 12 | 3+3 |
| SG14 | 72 | ADK | - | 20 | ND |

**Supplementary Table 2**

| **MAB** | **CLONE** | **REACTIVITY** | **FLUOROCROME** | **TARGET** | **SUPPLIER** |
| --- | --- | --- | --- | --- | --- |
| **CD184/CXCR4** | 12GS | Human | PE | C-X-C chemokine receptor type 4 | Miltenyi Biotec |
| **CD314/NKG2D** | REA1228 | Human | PE |  | Miltenyi Biotec |
| **Ang** | 14017.7 | Human | _ | Angiogenin | Abcam |
| **Angiop1** | - | Human | _ | Angiopoietin-1 | Abcam |
| **CD16/FcγRIII** | REA423 | Human | FITC | Fc-gamma-ReceptorIII | Miltenyi Biotec |
| **CD3** | BW264/56 | Human | PerCP | T-cell receptor (TCR) | Miltenyi Biotec |
| **CD49a** | REA1106 | Human | PE | α1 integrin | Miltenyi Biotec |
| **CD56** | REA196 | Human | APC | Neural cell adhesion molecule (NCAM | Miltenyi Biotec |
| **CD9** | REA1071 | Human | PE | Tetraspanin | Miltenyi Biotec |
| **CXCL12/SDF-1** | 79018 | Human | PE | C-X-C Motif Chemokine Ligand 12/Stromal cell-derived factor-1 | R&D SYSTEM |
| **CXCL8/IL8** | E8N1 | Human | PE | C-X-C Motif Chemokine Ligand 8/Interleukin-8 | Miltenyi Biotec |
| **GRZ-B** | CB9 | Human | PE | Granzyme-A | Miltenyi Biotec |
| **IFNγ** | 4S.B3 | Human | PE | Interferon-gamma | Miltenyi Biotec |
| **TNFα** | Mab11 | Human | PE | Tumor Necrosis Factor-alpha | Miltenyi Biotec |
| **VEGF** | # 23410 | Human | PE | Vascular Endothelial Growth Factor | R&D SYSTEM |
| **CD107a/LAMP-1** | H4A3 | Human | FITC | Lysosomal-Associated Membrane Protein 1 | BD Biosciences |
| **PD-1** | PD1.3.1.3 | Human | PE | Programmed Cell Death Protein 1 | Miltenyi Biotec |
| **TIM-3** | F38-2E2 | Human | PE | T-cell Immunoglobulin and Mucin domain 3 | Miltenyi Biotec |
| **CD45-FITC** | REA747 | Human | FITC | Cluster of differentiation 45, Pan-leukocyte | Miltenyi Biotec |
| **CD14-PE** | REA599 | Human | PE | Cluster of differentiation 14. | Miltenyi Biotec |

**Supplementary Table 3**

| **Human primers for qRT-PCR** | |
| --- | --- |
| ***Gene*** | ***Sequences*** |
| **hu 18s** | forward: *gcagaatccacgccagtacaag* |
|  | reverse: *gcttgttgtccagaccattggc* |
| **hu plgf** | forward: *tgtcaccatgcagctcctaa* |
|  | reverse: *gtctgtgggtctctgcttct* |
| **hu vegf** | forward: *ctgtcttgggtgcattggag* |
|  | reverse: *accagggtctcgattggatg* |
| **hu cxcl8** | forward: *cctgatttctgcagctctgtg* |
|  | reverse: *gtggtccactctcaatcactctc* |
| **hu vegfr2** | forward: *tctctctgcctacctcacct* |
|  | reverse:*accataccactgtccgtctg* |
| **hu cxcl12** | forward: *ctcaacactccaaactgtgccc* |
|  | reverse: *ctccaggtactcctgaatccac* |
| **hu pai** | forward: *tctctgccctcaccaacatt* |
|  | reverse: *cggtcattcccaggttctct* |
| **hu icam-1** | forward: *agcggctgacgtgtgcagtaat* |
|  | reverse: *tctgagacctctggcttcgtca* |
| **hu vicam-1** | forward: *gattctgtgcccacagtaaggc* |
|  | reverse: *tggtcacagagccaccttcttg* |
| **hu cxcr4** | forward: *ctcctctttgtcatcacgcttcc* |
|  | reverse: *ggatgaggacactgctgtagag* |
| **hu il-1α** | forward: *tgtatgtgactgcccaagatgaag* |
|  | reverse: *agaggaggttggtctcactacc* |
| **hu arg1** | forward: *gattctcagtgctgcggatc* |
|  | reverse: *cagcttctcttatggcagcg* |
| **hu il10** | forward: *gccaagccttgtctgagatg* |
|  | reverse: *aagaaatcgatgacagcgcc* |
| **hu il12** | forward: *gccttcaccactcccaaaac* |
|  | reverse*: atggtaaacaggcctccact* |
| **hu cd206** | forward: *agccaacaccagctcctcaaga* |
|  | reverse: *caaaacgctcgcgcattgtcca* |
| **hu tgf-β** | *forward: ctgtccaacatgatcgtg* |
|  | *reverse: cgcagtcctctctccatc* |
| **hu cd80** | *forward: ctcttggtgctggctggtcttt* |
|  | *reverse: gccagtagatgcgagtttgtgc* |
| **hu cd86** | *forward: ccatcagcttgtctgtttcattcc* |
|  | *reverse: gctgtaatccaaggaatgtggtc* |
